# Annotations

**Published:** 1899-06-03

**Authors:** 


### Annotations.

### A Finger in Every Pie.

At a recent meeting of the Metropolitan Radical Federation the following interesting resolution was adopted unanimously: "Inasmuch as the tendency is more than ever among all Democrats towards the placing of all those institutions supported in any way by the people's money directly under public representative control, in our opinion the time has now arrived when all hospitals and dispensaries should be maintained out of public funds and administered by the representatives of the people." We are very pleased to see the interest shown by the Metropolitan Radical Federation in the hospitals and dispensaries from which their *clientèle* derive such untold benefits, but we confess we do not see the connection between the first half of their resolution and the second. There are certain hospitals and dispensaries which are supported "by the people's money," or, at least, by the money of such of "the people" as pay rates, but they are already administered by the representatives of people so far as the Poor Law Guardians deserve that title; and if the desire of the Radical Federation is merely that a larger proportion of "the people" should share in the paying of rates and in the administration of rate-supported hospitals, we can only commend their public spirit. But they clearly are quite in error in imagining that the voluntary hospitals of London are supported by "the people's money," as also they are if they think that there is no representative control over these hospitals by those who subscribe the money by which they are supported. If a man puts a penny or a £20 note into a collecting-box and walks away, of course he gets no representation; but if he subscribes regularly to a hospital, whether he does it directly or through a club, he generally can have his share, through his representative, of course, in the management of the charity. This is representative control.

### The Progress of the Plague.

The announcement that plague has established itself in Egypt is a matter of the greatest concern to Europe at large, for it shows that whatever may be the deeper causes of that tendency to extend into new countries which plague has exhibited during recent years they are by no means limited to those regions in India and the far East where the disease has been so fatally prevalent. As it seems to us, there is no advantage to be gained in pretending to an accuracy and fulness of knowledge on the subject which we do not possess, and confidently as some journals may choose to speak of plague as a disease which can be more effectually dealt with and stamped out than perhaps any other, we can have little hesitation in asserting that we remain to this day quite ignorant of the causes of the rapid recession of the disease from western Europe after what we are in the habit of speaking of as the "great plague" in London in 1664, and that we are equally ignorant in regard to the causes of the recrudescence of the disease which first showed itself to the outside world when it broke out in Hong Kong some five years ago. Nor can we admit that the history of the disease in India gives any good ground for believing that the measures adopted for controlling its progress have had any very decided effect, except in so far as they have made for improved sanitation and have led to some diminution of the terrible dirt and overcrowding which undoubtedly act as the most fertile among the predisposing causes of the disease. Again and again it has happened that just as we were pluming ourselves on the success of the measures taken for its prevention the whole thing has cropped up again just as badly as before; and, as an almost equally disheartening event, epidemics have in other cases died away coincidently with the cessation of attempts at inspection and segregation and other favourite methods for their suppression. Roughly, we know that plague is in some way connected with filth and insanitation, with rats, and fleas, and bugs, and other forms of biting vermin, with overcrowding and want of light and air, and with the state of body which results from the use of innutritious food; and when we see that in Eastern countries, however much the natives may suffer, the English mostly escape attack, we are apt to feel that Europe need not fear. But the English who thus escape are the cleanly and well-fed English, not the English of the slums; and much as we may hope from the general measures of sanitation which during the past twenty years have so greatly changed the conditions of life in most northern capitals, so far as public sanitation is concerned, we know too well what are the conditions of the dwellings which serve as homes for thousands—nay, for millions—of the people of this continent, to feel any confidence that Europe is proof from an attack of plague. As we have just said, the conditions in the midst of which, as all history shows, plague is apt to occur are well enough known, however little we may be able to demonstrate the part which each of them plays in its propagation. And who will assert that the conditions which we have enumerated do not exist in London—here at our very doors?

### The Sale of Sanitary Indulgences.

A recent case in which the sanitary bye-laws of the London County Council have been set at naught in the construction of a great hotel, and in which the whole affair seems to have been condoned by the infliction of a small penalty in a police court, is a matter of very serious importance in that it suggests to all who have to do with the building trade that the regulations laid down in regard to the planning of new buildings may be defied with comparative impunity if only it can be shown when the case comes before the magistrate, that it would be a very expensive affair to put things right. When the builders of a great hotel were recently summoned by the sanitary authority for having put nine soil pipes inside the building instead of outside, as required by the bye-laws of the London County Council, it was argued that if the situation of the soil pipes complained of were to be altered, the structure would have to be half pulled down; that it would be unreasonable to compel the defendant to half pull down a structure which had cost nearly £500,000 just to alter the drains; and that a fine would meet the case. Curious to relate, the vestry seemed to see no objection to this course, and in the end a fine of £2 was imposed. The sale of indulgences was one of the things which led up to the reformation in times gone by, and perhaps reformation in vestries may follow so obvious an abuse as this. Two pounds for nine sanitary sins does not seem dear.

